# Short-stem total hip arthroplasty is not associated with an earlier return to work compared to a straight-stem design

**DOI:** 10.1038/s41598-021-82805-0

**Published:** 2021-03-02

**Authors:** Georg Hauer, Maria Smolle, Sabrina Zaussinger, Joerg Friesenbichler, Andreas Leithner, Werner Maurer-Ertl

**Affiliations:** Department of Orthopaedics and Trauma, Medical University of Graz, Auenbruggerplatz 5, 8036 Graz, Austria

**Keywords:** Outcomes research, Orthopaedics

## Abstract

Return to work (RTW) has been specifically identified as a high priority in patients undergoing total hip arthroplasty (THA). This investigation sought to assess the effect of the stem design on patients’ RTW. Secondly, the study aimed to identify risk factors that lead to a delayed RTW. Questionnaires inquiring about RTW, employment history, educational level, type of work, physical demands and joint awareness were administered by post. Further data were collected from patients’ hospital records. 176 patients who underwent THA using a short-stem and 97 patients using a straight-stem design were compared. The median return to work time was 10 weeks [IQR 7–14 weeks], with no significant difference between the two groups (short stems vs. straight stems; 10 [IQR 7–14] vs. 11 [7.5–13.5] weeks; p = 0.693). In the multivariate linear regression analysis, self-employment vs. employee (p = 0.001), dimension of preoperative workload (p = 0.001), preoperative sick leave (p < 0.001), and hospital length of stay (LOS) (p < 0.001) independently affected the period until work was resumed. The Forgotten-Joint-Score-12 showed no significant difference between the two groups. The data show that the majority of THA patients can expect to resume work and stem design has no impact on RTW. Employees with preoperative sick leave, prolonged hospital LOS and low workload are at higher risk for a delayed RTW.

## Introduction

Among the increasing number of total hip arthroplasty (THA), younger patients have been responsible for the most rapid rate of growth^1^. THA is no longer an intervention of the elderly, but also affects the working-age population^2^. As this trend is set to continue, the ability of THA to facilitate a return to work (RTW) is becoming increasingly important.

Owing to the fact, that younger THA receivers are at a higher risk for implant failure, it is essential to provide more native bone during primary surgery should revision surgery become necessary^3^. In recent years, there has been an increasing interest in short-stem prostheses, which have shown to be able to preserve femoral bone stock and to decrease periprosthetic bone losses secondary to stress shielding^4–6^. Encouraging results led to the expansion of indications of short-stems, however, despite a strong theoretical basis for their use, there is still some uncertainty that needs to be clarified before short-stem THA has a definite role to play in modern THA.

The majority of patients who are employed before a hip arthroplasty return to work after the surgery^7^. However, there is a lack of information in the literature to provide to patients about their RTW after short-stem THA. Therefore, the primary study aim was to examine the impact of short-stems on RTW compared to a conventional straight-stem design. Secondly, the investigation aimed to identify risk factors of delayed RTW after THA among factors related to demographic variables, work situation and socioeconomic status.

## Material and methods

A retrospective review of all patients that underwent elective THA between November 2013 and August 2019 was performed using the local hospital database. Those who were below the retirement age (male < 65, female < 62) at the time of their THA, had a diagnosis of non-inflammatory arthritis (osteoarthritis, osteonecrosis, developmental dysplasia) and were at least 6 months post-surgery were included. Exclusion criteria were applied to individuals who were fewer than 6 months post-surgery, had a one-stage bilateral THA or hip replacement due to a femoral neck fracture or a tumour.

There were a total of 581 THA patients who met the inclusion criteria. Of these, 337 received short-stem THA and 244 straight-stem THA. The decision to either perform a short-stem or straight-stem THA was based on the digital preoperative planning, the surgeon’s preference and the intraoperative decision based on the characteristics of the femur, including bone quality. In all short-stem patients, the cementless, metaphyseal-anchoring, femoral-neck preserving *Ana Nova Alpha Shaft Proxy (ImplanTec)* was used. All patients in the straight-stem group received a cementless *Corail* stem *(Johnson&Johnson).* Both stems were combined with cementless press-fit cups *(Ana Nova Alpha Cup, ImplanTec* or *Pinnacle Cup, Johnson*&Johnson) with a ceramic-ceramic bearing couple. All operations were performed using the minimally invasive, muscle-sparing, modified anterolateral approach in a standardised manner. The procedures were performed or supervised by senior hip surgeons who were all considered to have overcome their operative learning curve. Postoperatively, both groups followed an identical standardized rehabilitation protocol that consisted of full weight bearing immediately after surgery with the use of crutches.

Patients who worked were allowed to RTW as soon as they were comfortable doing so. Patients were also encouraged to resume activities as soon as tolerated.

Following appropriate patient inclusion, all patients received a questionnaire and a consent form describing the study aims by letter. The questionnaire contained items related to the level of education and the profession, employment status and weekly hours of work pre- and post-operatively, preoperative sick leave related to the hip, the length of time taken to resume work, the characteristics of their job, patients’ satisfaction with the length of time until the RTW and patients’ awareness of their artificial joint. The time to RTW was defined as the length of time from the patients’ surgery to their return to any amount of work.

**Figure 1:**
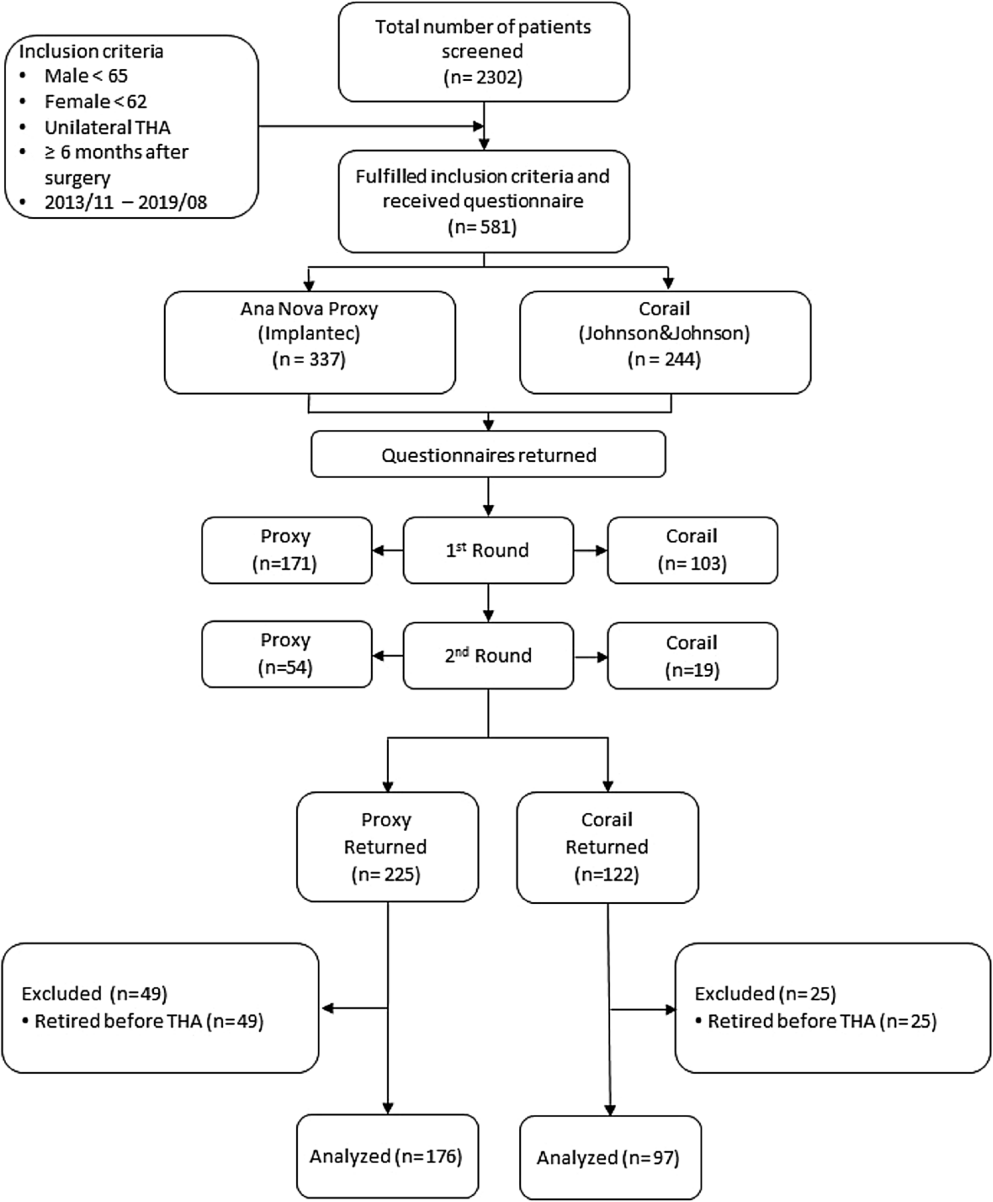
Patient flow chart on study inclusion.

A questionnaire was posted to those patients who fulfilled the inclusion criteria. A reminder call was made to those patients who did not return the questionnaires within 6 weeks. If there was no response for another 6 weeks, they were excluded. Responses from 347 patients and no response from 234 patients were received. From these 347 returned questionnaires, another 74 patients who replied that they were already retired at the time of surgery had to be excluded. A patient flow chart on study inclusion is given in Fig. 1.

The data of each patient were collected, both the patient’s completed questionnaires and a review of the patient’s medical record. The data collected from the medical record included age, sex, body-mass index (BMI), the site of surgery, hospital length of stay (LOS), type of stem and previous joint replacements. Furthermore, the Forgotten Joint Score-12 (FJS-12) was applied to assess patients’ awareness of their artificial joint in everyday life. The final score range is 0 (worst) to 100 (best)^8^.

All methods were carried out in accordance with relevant guidelines and regulations. The study was approved by the local ethics committee of the Medical University of Graz, Austria (IRB-No. 31-453 ex 18/19). A written informed consent was obtained from all participants.

### Statistical analysis

Responses to the questionnaires were entered into a database and statistical analyses were performed with Stata Version 13.0 (StataCorp, College Station, US). Differences for categorical variables between the groups were assessed with chi-squared tests. Wilcoxon-rank-sum-tests and (paired) t-tests were performed to analyse differences between groups for non-parametric and parametric variables, respectively. The International Classification of Occupations (ISCO) was used to classify the patients’ current occupations into ten hierarchical categories based on the duties and responsibilities undertaken in the job^9^. For statistical analyses, non-manual workers (ISCO groups 1–5) and manual workers (ISCO groups 6–9) were analysed separately.

Logistic regression was performed to assess influence of continuous parameters on binary variables, providing corresponding odds ratios (OR), 95% confidence intervals (95% CIs) and p-values. Univariate as well as multivariate linear regression analyses were performed to estimate the influence of parameters on the RTW time, after excluding those patients who had planned to retire following surgery and those who had actually retired. For the multivariate analysis, a stepwise backward selection model was constructed, initially including all variables significant in the univariate analysis. With the stepwise backward approach, variables were subsequently eliminated from the full model in case of a p-value of > 0.05 until a model explaining the data best, had been found. For all analyses, a p-value of < 0.05 was considered statistically significant. Power analysis was performed for the multivariate linear regression analysis using Stata’s power rsquared command.

## Results

### Descriptive analysis

Of the 273 patients included, 176 received a short-stem (*Proxy*; 64.5%) and 97 a straight-stem THA (*Corail*; 35.5%). The mean patient age at surgery was 53.9 years (SD: 6.2 years), with no significant difference between groups (p = 0.403). Mean patient age at time of questionnaire was 56.0 years (SD: 0.4 years), with a significant difference between groups (*Proxy* vs. *Corail*; 55.1 ± 0.5 years vs. 57.7 ± 0.6 years; p = 0.001. Correspondingly, mean time from THA to completion of the questionnaire was 30.9 months (± 20.2 months), with a significantly shorter interval for patients with a short-stem THA in comparison to a straight-stem THA (20.5 ± 10.7 months vs. 50.0 ± 19.4 months; p < 0.001). 128 patients were female (46.9%) and 145 male (53.1%), again with no significant difference between patients having received either short-stem or straight-stem THA (p = 0.161). Furthermore, there was no significant difference between the two groups in terms of BMI (p = 0.810) or previous endoprosthetic replacements (p = 0.199; Table 1). The mean postoperative FJS-12 was 67.8 ± 24.9 with no significant difference between short- and straight-stem groups (69.4 ± 24.0 vs. 64.9 ± 26.4; p = 0.162).

**Table 1:** Patient characteristics of both groups and median delay in RTW following THA.

	Short-stem (Proxy)	Straight-stem (Corail)	Missing (n)	p-value
Age at surgery (in years)	54.2 ± 6.4	53.5 ± 5.8	0	0.403^a^
**Gender**				
Male	99 (56.3%)	46 (47.2%)	0	0.161^b^
Female	77 (43.7%)	51 (52.6%)		
BMI	27.9 ± 5.6	28.1 ± 5.7	0	0.810^a^
**ASA**				
1	56 (32.0%)	19 (19.6%)	1	**0.017**^b^
2	102 (58.3%)	59 (60.8%)		
3	17 (9.7%)	19 (19.6%)		
**Already another endoprothesis**				
No	155 (88.6%)	78 (83.0%)	4	0.199^b^
Yes	20 (11.4%)	16 (17.0%)		
Hospital length of stay (days)	6.3 ± 1.8	7.0 ± 1.4	0	**0.006**^**a**^
Return to work (weeks) (median)	10 [IQR 7–14]	11 [7.5–13.5]	6	0.693^c^

Bold values indicates statistical significance at the p < 0.05 level.

*IQR* interquartile range.

^a^t-test.

^b^Chi-squared test.

^c^Wilcoxon-rank-sum-test.

### Return to work

**Figure 2:**
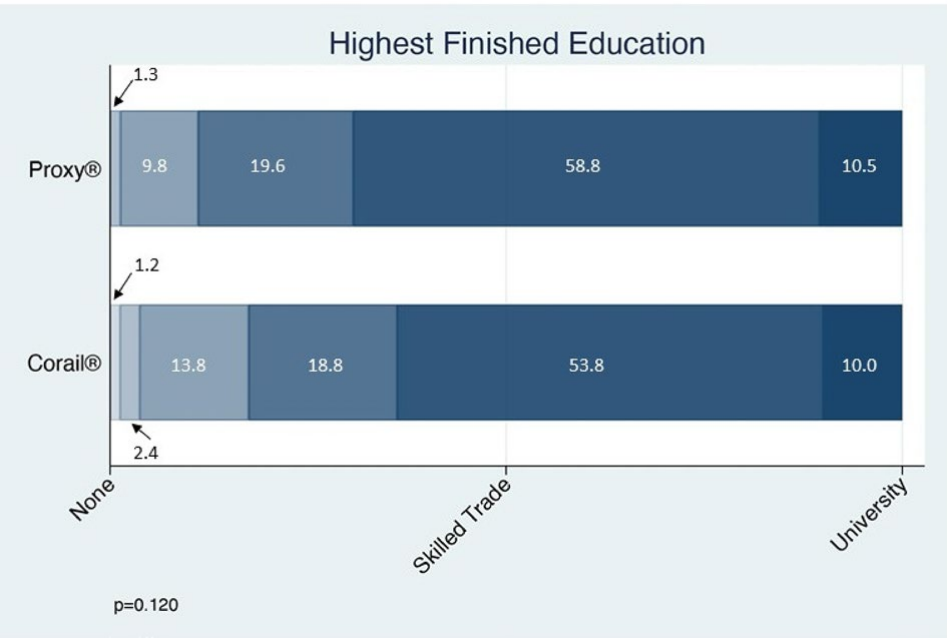
Highest finished education by study group.

Before surgery, 141 patients in the short-stem group (83.4%) and 72 patients (78.3%) in the straight-stem group were employees and the others were self-employed, whilst information was missing on 12 patients (p = 0.303). Eleven (6.4%) and 13 patients (13.4%) in the short- and straight-stem group, respectively, were currently unemployed (p = 0.051). Furthermore, 62 (35.4%) and 34 (36.6%) patients in the short- and straight-stem groups had been on sick leave preoperatively because of complaints with their hip (p = 0.854). There was no significant difference in the highest education attained between the two groups (p = 0.120; Fig. 2).

There was also no significant difference between the patients’ current occupation according to ISCO groups “non-manual workers” and “manual workers” (63.0% and 40.0% vs. 53.8% and 46.2% in short- and straight-stem groups, respectively; p = 0.143).

Following surgery, 164 (94.8%) and 78 patients (83.0%) of the short- and straight-stem groups, respectively, resumed work, whilst 6 (3.5%) and 3 (3.2%) did not return to work, and three (1.7%) and 13 (13.8%) retired (p < 0.001). The median delay in RTW following THA was 10 weeks [IQR 7–14 weeks], with no significant difference between the two groups (short stems vs. straight stems; 10 [IQR 7–14] vs. 11 [7.5–13.5] weeks; p = 0.693).

### Factors associated with a delayed return to work

In the univariate linear regression analysis (excluding all patients who had already retired at time of surgery or planned to retire following surgery), the time interval from the THA to the completion of the questionnaire was not significantly associated with the time stated by patients until returning to work (F(1, 234) = 2.11; p = 0.148). Furthermore, there was no influence of BMI (F(1, 234) = 0.248; p = 0.117), stem design (F(1, 234) = 0.01; p = 0.932), previous endoprosthesis (F(1, 232) = 0.01; p = 0.925), ISCO group (F(1, 231) = 0.05; p = 0.823), preoperative diagnosis (osteoarthritis, avascular necrosis, hip dysplasia; F(1, 233) = 1.41; p = 0.245) and highest finished education (F(1, 196) = 0.99; p = 0.322) on the RTW. Female gender (F(1, 234) = 10.38; p = 0.001), prolonged hospital LOS (F(1, 234) = 6.03; p = 0.015) and preoperative sick leave (F(1, 232) = 14.02; p < 0.001) were significantly associated with a delayed RTW in the univariate setting. On the other hand, in the univariate setting, self-employment (F(1, 225) = 18.47; p < 0.001), increased preoperative workload (F(1, 229) = 26.67; p ≤ 0.001), young age at surgery (F(1, 234) = 6.46; p = 0.012) as well as young age at questionnaire (F(1, 234) = 9.69; p = 0.002) were significantly associated with a shortened RTW.

**Figure 3:**
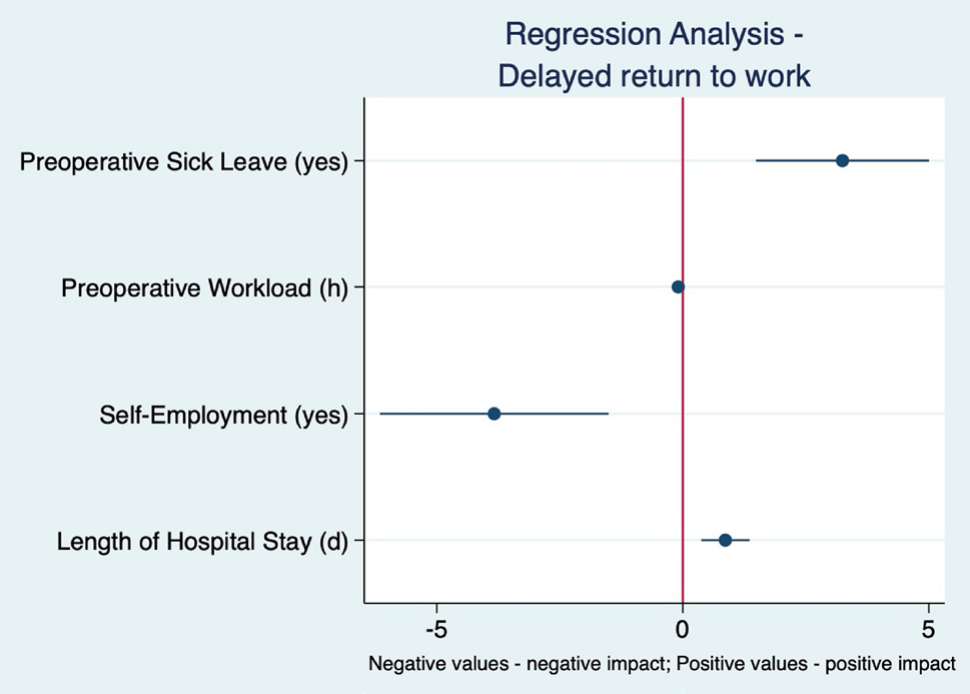
Graphical presentation of multivariate linear regression analysis assessing effect of variables on time to resume work following THA.

Consequently, in the multivariate linear regression analysis (after excluding age at surgery (p = 0.053), gender (p = 0.534) and age at questionnaire (p = 0.414)), the factors that independently affected the time period until work resumption were the type of preoperative occupation (self-employment vs. employee) (Coef.: − 3.39; p = 0.001), dimension of the preoperative workload (Coef.: − 0.10; p = 0.001), preoperative sick leave (Coef.: 3.32; p < 0.001), and hospital LOS (Coef.: 0.83; p < 0.001 (Table 2, Fig. 3). Thus, all four factors significantly predicted the time to work resumption (F(4, 216) = 20.94; p < 0.0001; R2 = 0.279; 1 − β = 1.00).

**Table 2:** Multivariate linear regression analysis assessing the variables influencing the time period to work resumption.

	Coefficient	SE	95% CI [Lower; Upper]	p-value^a^
**Preoperative sick leave**				
No	Ref			**< 0.001**
Yes	3.32	0.81	[1.74; 4.91]	
Preoperative workload (h)	− 0.10	− 23.39	[− 0.17; − 0.04]	**0.001**
**Preoperative occupation**				
Employee	Ref			**0.001**
Self-employment	− 3.39	1.04	[− 5.44; − 1.33]	
Hospital length of stay (days)	0.83	0.22	[0.39; 1.26]	** < 0.001**
Constant	13.19	2.50	[8.26; 18.13]	** < 0.001**

Bold values indicates statistical significance at the p < 0.05 level.

*Excluded variables* gender (p = 0.534), age at surgery (p = 0.053), age at questionnaire (p = 0.414).

*SE* standard error, *CI* confidence interval.

^a^Multivariate linear regression analysis.

### Patients’ satisfaction with their RTW time

Overall, 95.5% of patients had been satisfied or very satisfied with RTW time, whilst 4.5% of patients were unsure or dissatisfied, with no significant difference between stem groups (short stem vs. straight stem: 97.0% vs. 92.2% satisfied or very satisfied; p = 0.093). Moreover, according to logistic regression, neither age at surgery (OR 0.936; 95% CI 0.866–10.011; p = 0.092), nor age at questionnaire (OR 0.945; 95% CI 0.875–1.020; p = 0.149), or time interval from surgery to questionnaire (OR 1.012; 95% CI 0.984–1.041; p = 0.411) had a significant influence on patients’ satisfaction with regards to RTW time.

## Discussion

The most important finding was that almost 90% of the patients were able to RTW after hip replacement surgery. This is essential, since a successful RTW has been identified as a crucial outcome marker for patients after THA^7,10^. According to the present study, short-stems did not reduce the RTW time when compared with straight-stem THA. This can be interpreted as stem design has no impact on the recovery time.

THA is an increasingly popular treatment option, especially within the working-age population^1,11^. Several studies in the literature have reported on RTW after THA but none of them provided a comparison between two different stem designs.

In this study, the median delay in RTW following THA was 10 weeks with no significant difference between the short-stem and straight-stem groups. The RTW time of this study is in line with previous studies. RTW after THA ranged from 1 to 10 weeks after surgery and the post-surgical proportion of patients that returned to work ranged from 25 to 95%^7,12–17^. Considering these results, the majority of patients employed before their THA resumed work postoperatively.

In the current investigation, the method of a standardised minimally invasive surgical approach was identical for all patients. We believe that a soft-tissue and muscle sparing approach in combination with immediate postoperative weight bearing as soon as tolerated are primarily responsible for a quick recovery and caused the comparable time to RTW between the short- and straight-stem designs. It has already been shown that the application of minimally invasive surgery techniques during THA has helped reduce the patients’ pain and has accelerated recovery. Patients who had minimally invasive THA did not take as long to be able to sit up, stand and resume their daily commute which allowed the patients to return to work sooner^18–20^.

It is important to identify individuals who are at risk of a delayed RTW. A safe RTW can often be ensured by the awareness of modifiable risk factors and may help with appropriate patient selection^21,22^. This study corroborates previous findings that sick leaves before the surgery are an important determinant of RTW after hip replacement surgery^13,14^. In this study, preoperative sick leave appears to have a negative impact on the length of time before the RTW after surgery. Kleim et al.^13^ found that patients who had preoperative sick leave due to their hip or knee arthritis took 4.6 weeks longer to RTW than those who did not. It would therefore be reasonable, to suppose that the working-age population should have minimum preoperative sick leave in order to maximize the chances of regaining postoperative employment^16^.

In the present study, the factor with the greatest effect on shortening the patients’ time to RTW was being self-employed. This finding is in line with Styron et al.^23^, but does not reflect the observations from other investigators. Mobasheri et al.^16^ stated that being self-employed rather than a salaried employee did not affect the time to RTW postoperatively. Bohm^24^ also did not observe an association between being self-employed and the RTW. Therefore, it remains unclear if self-employment is an important determinant of the RTW.

Interestingly, shorter hospital LOS had a significantly positive effect on the RTW. It is known that preoperatively optimized patients in terms of modifiable aspects of their health have shorter LOS after primary hip arthroplasty surgery^25,26^. It is therefore likely that healthier patients have a shorter hospital LOS thus a lower risk for an unsuccessful RTW after THA. This is supported by findings from Cowie et al.^12^ and Bohm^24^, who also found a correlation between the RTW and the presence of comorbid diseases and the patient’s physical condition.

Within this study, the preoperative workload in hours was another predictor of the RTW after THA. Findings suggest that motivation plays a role in the time taken to RTW^23^. It is likely that people with higher workloads have motivated character traits and therefore tend to work a lot. On the contrary, while several authors reported a correlation between the educational level and the RTW, education was not a determinant of early RTW in the present analysis^13,23^. This was a surprising finding, since motivational factors certainly also apply to people with a further education. The influence of motivation to RTW, although not specifically assessed in this study, remains unknown to a certain extent.

It is reasonable to expect that a job with higher physical requirements would result in longer time to RTW, however, the spectrum of occupational grades according to ISCO was not significantly associated with the time stated by patients until their RTW. It appears that properly managed, patients are capable of returning to work in physically demanding jobs^23^. Contrary to these findings, Laasik et al.^14^, in a nationwide cohort study, found that patients with non-manual occupational status had a higher probability of RTW than manual workers. There is also conflicting evidence regarding the time to RTW and its association with age, gender and BMI. In the present univariate analysis, age and gender both had a significantly effect on the RTW. In multivariate analysis, however, no gender and age-related differences were observed. BMI was also not found to have a statistically significant relationship with RTW. The current results partly agree with previous reports on the one hand, but are also in contrast to other findings^7,12,14,15,17,22^. Nevertheless, all modifiable potential risk factors should be accounted for when planning THA. Even though the BMI might not be a definitive determinant of RTW, obesity has clearly been demonstrated to be an independent risk factor for an increased risk for complications after THA^27–29^.

The authors acknowledge limitations in this investigation. The reason for some differences between the results of the present and previous studies remain unclear. However, there are marked differences in work practices, welfare and pension arrangements, retirement ages and cultural attitudes towards work between countries. Therefore, the present sample is not representative for all populations. The next point is that data were collected in a retrospective manner, and results should be interpreted accordingly. A randomised-controlled study design would be beneficial to confirm the present findings. Another limitation is the inconclusive response rate (59.8%), which could represent a selection bias. Patients who are still unemployed or still on sick leave may not have responded. Furthermore, the discrepancy in age at questionnaire was due to the different time periods, in which the two hip systems had been used. Short-stems became increasingly popular since 2016 and now make up the majority of all hip replacements in our institution. Another weakness of the study was that some patients were contacted up to 6 years postoperatively and their recollection of events and in particular their time to return to work, may not be entirely reliable^30^.

In conclusion, the postoperative RTW is multifactorial and most patients can expect to RTW within a short time. Stem design may not play an important role when thinking about the working-age population and returning them to work successfully. Employees with preoperative sick leave, prolonged hospital LOS and low workload are at higher risk for a delayed RTW. The findings from this study are valuable for educating patients regarding recovery timeline after short- and straight-stem THA.

## Supplementary Information

Supplementary Information.

## Data Availability

Data are available upon reasonable request.
