# Supplementary material for: Short-stem total hip arthroplasty is not associated with an earlier return to work compared to a straight-stem design
Source: Sci Rep. 2021 Mar 2;11:4968. doi: 10.1038/s41598-021-82805-0 (PMC7925530; doi:10.1038/s41598-021-82805-0)
Supplement: Supplementary file 1 — Supplementary Information. [file 41598_2021_82805_MOESM1_ESM.docx]

**Short-stem total hip arthroplasty is not associated with an earlier return to work compared to a straight-stem design.**

Georg Hauer^1^, MD*, Maria Smolle^1^, MD, Sabrina Zaussinger^1^, Joerg Friesenbichler^1^, MD, Andreas Leithner^1^, MD, Werner Maurer-Ertl^1^ MD*

^1^ Department of Orthopaedics and Trauma, Medical University of Graz, Graz, Austria

***Correspondence:** Dr. Georg Hauer & Dr. Werner Maurer-Ertl

Department of Orthopaedics and Trauma

Medical University of Graz

Auenbruggerplatz 5

Graz A-8036, Austria

Email: georg.hauer@medunigraz.at;

werner.maurer-ertl@medunigraz.at

Phone: +43 316 385 81198

Appendix 1 –Questionnaire for Patients

***Preoperative Questionnaire for Patients***

1. In the 6 months before surgery, did you work for pay at all?

- Yes
- No
  1. If No, was this primarily due to your hip pain?
- Yes
- No

1. How many hours did you work before your hip surgery?

_____________________hrs/week

1. Before your surgery, did you have to take sick leave because of your hip?

- Yes
- No
  1. If Yes, how many weeks of sick leave was it in total?
     _____________________weeks

1. In the last job before surgery, were there any options to make changes in your job to relieve your hip pain?

- Yes
- No
  1. If Yes, please describe:

1. Were you employed or were you self-employed?

- Employed
- Self-employed

1. What is the highest level of education you completed?

- Nothing
- Less than High School
- Vocational/Trade/Technical School
- Some High School
- High School with Degree
- University or higher

1. What was your job before surgery? Please describe.
   _________________________________________
2. Please describe your intention after surgery?

- Return to the same job
- Return to any job
- I don’t want to work again
- Retirement

1. If “0” is no pain, and “10” is the worst pain you can imagine, where was your pain before surgery?
   0 – 1 – 2 – 3 – 4 – 5 – 6 – 7- 8 – 9 – 10

***Postoperative Questionnaire for Patients***

1. Since your surgery, have you worked at all?

- Yes
- No
- Retirement
  1. If No, did you not return because of your hip?
- Yes
- No
  1. If No, please explain.

1. How many weeks did it take you to start working again?
   ____________________weeks
2. After your surgery, for how many hours per week were you able to work?
   _____________________hrs/week
3. After your surgery, did you return to your usual job you had before surgery?

- Yes
- Yes, but different company/organization
- No

1. Are you satisfied with the length of time until you could start working again?

- Very satisfied
- Satisfied
- Unsure
- Dissatisfied
- Very dissatisfied

1. What do you think about the length of time after your hip surgery before you could work again?

- I would have thought it would take longer.
  How long? __________________weeks
- It is exactly as I expected it to be.
- I would have thought it would be faster.
  How much faster? ___________________weeks

1. If “0” is no pain, and “10” is the worst pain you can imagine, where is your pain now after surgery?
   0 – 1 – 2 – 3 – 4 – 5 – 6 – 7- 8 – 9 – 10
